# Historical Overview and Pathologization of Sexual and Gender Diversity in Brazilian Psychological Assessment

**DOI:** 10.1007/s12124-026-09973-4

**Published:** 2026-03-09

**Authors:** Angelo Brandelli Costa, Silvia Helena Koller, Henrique Caetano Nardi

**Affiliations:** 1 Psychology Graduate Program, Pontifical Catholic University of Rio Grande do Sul, Porto Alegre, Brazil; 2 Department of Psychological and Social Sciences, John Cabot University, Rome, Italy; 3 North West University - South Africa, Potchefstroom, South Africa; 4 Social Psychology Graduate Program, Federal University of Rio Grande do Sul, Porto Alegre, Brazil

**Keywords:** Psychological assessment, Gender bias, Sexual diversity, Brazilian psychology

## Abstract

This article analyzes personality assessment instruments used in Brazilian psychology in the early 2000s. It seeks to understand how tests that may contain discriminatory biases related to sexual and gender diversity coexisted with guidelines that defend human rights and the elimination of prejudice. The historical evolution of gender and sexuality assessment in psychology is explored, from the 18th-century concept of “single sex” to the development of psychological tests that measure “masculinity” and “femininity”. The article examines the ethical and technical implications of using such instruments, highlighting their potential discriminatory biases and contradictions with ethical guidelines. Historical influences on these instruments are addressed, including evolutionary and psychoanalytic theories, as well as the development of personality tests. The shift from the pathologization of gender and sexuality variations to the recognition and affirmation of diversity is also discussed. The article highlights a paradox in Brazilian psychology, where regulatory bodies emphasize ethical principles and human rights, yet historically approved and maintained tests with clear discriminatory biases. This is attributed to a historical disconnect between empirical psychology and critical/feminist psychology in Brazil, unlike the more integrated approach in the North American context where the tests were developed.

This article aims to analyze the Factorial Personality Inventory (IFP) (Pasquali et al., 1997) and the Comrey Personality Scales (CPS) (Costa, 2009) within the context of Brazilian psychology in the early 2000s. The goal is to understand how these instruments, with potential discriminatory biases concerning sexual and gender diversity, coexisted with resolutions from the Brazilian Federal Council of Psychology (CFP) that advocated for human rights and the elimination of prejudice against sexual diversity and the promotion of gender equality. The discussion presented in this article had already been published on two other occasions, in Portuguese, for the Brazilian context (Costa & Nardi, 2013; Costa et al., 2017), and is now presented to an international audience. These two specific instruments were selected for this analysis for strategic reasons. First, during the early 2000s, both the IFP and the CPS were among the most widely used personality assessment tools in Brazil, frequently employed in high-stakes contexts such as personnel selection and public service entrance exams. Second, and most importantly, they serve as the clearest paradigmatic examples of the tension between psychometric stability and ethical obsolescence. Unlike other tests that may have had subtle biases, the IFP and CPS explicitly contained scales labeled ‘Heterosexuality’ or ‘Masculinity/Femininity’ based on outdated theories, making them the ideal case studies to illustrate how discriminatory biases remained technically approved within the Brazilian regulatory framework.

The Sistema de Avaliação de Testes Psicológicos (SATEPSI), or Psychological Test Assessment System, is a crucial regulatory framework established by the Conselho Federal de Psicologia (CFP) in Brazil. Instituted in 2001 through Resolution CFP nº 025/2001 and subsequently updated, with the current iteration being Resolution CFP nº 31/2022, SATEPSI’s primary objective is to ensure the technical-scientific quality and ethical utilization of psychological tests within professional practice. This system serves as a comprehensive registry, categorizing psychological instruments as “favorable” (i.e., approved for use based on empirical evidence of validity and reliability) or “unfavorable.” By providing this authoritative guidance, SATEPSI safeguards the integrity of psychological assessment, protects the public from the misuse of unvalidated instruments, and promotes adherence to rigorous scientific standards in Brazilian psychology (Reppold & Noronha, 2018). Only tests approved and listed in this system can be used by Brazilian psychologists.

Among the instruments listed was the Factorial Personality Inventory (IFP) (Pasquali et al., 1997). This instrument aims to evaluate individuals across 15 psychological needs, namely: succorance, dominance, order, abasement, intraception, achievement, exhibition, nurturance, change, endurance, aggression, deference, autonomy, affiliation, and heterosexuality, with items such as “I like to kiss attractive people of the opposite sex” and “I like to get sexually aroused.” Each need comprises a nine-item scale to be rated on a 7-point Likert-type scale ranging from “1 = Not at all characteristic” to “7 = Totally characteristic.” Additionally, the test has a social desirability scale (12 items) and a lie or validity scale (8 items), totaling 155 items. The Brazilian adaptation was carried out by Pasquali et al. (1997) with a sample of 3,399 subjects from 11 Brazilian states. The instrument was evaluated and approved for use by SATEPSI (Psychological Test Assessment System) in 2003, remaining available for use until 2018.

The Comrey Personality Scales (CPS), originally published in 1970 by American psychologist Andrew Comrey (Comrey, 1970), had its first Brazilian version published in 1973 (Rodrigues, 1979). Revised in 2003, its third edition (Costa, 2009) also remained available for use in Brazil until 2018. It is a test that focuses on the assessment and description of “normal” personality, but “it can also be useful in identifying psychiatric problems or those requiring psychotherapeutic interventions” (Costa, 2009, p. 15). The instrument consists of 100 statements distributed across 10 subscales, aimed at evaluating different personality factors. Each statement is rated on a 7-point scale, ranging from “never (certainly not)” to “always (certainly yes).” The average of responses to the items forming each subscale is interpreted by an axis of values that directs the extremes of each factor. Among the subscales is that of Masculinity and Femininity (M). Regarding it, the author states that “individuals with high scores on this factor reported being ‘strong,’ stubborn, and tough, not easily impressed by violent scenes, tolerating vulgarities, and not crying easily or showing interest in romantic stories and love” (p. 64). He also states that “those with low scores cry easily, are bothered by the sight of insects and reptiles, and showed interest in romantic stories” (p. 64). Although the review’s author expresses that the CPS does not aim to identify heterosexuality or homosexuality, he also states that “the incidence of some values in the extreme ranges, upper or lower, points to the possibility of pathological social conduct impairments” (p. 75). The author of the original instrument states that “high scores on the M scale were statistically associated with women with homosexuality and in men with a diagnosis of schizoid personality and problems with the law” (Comrey, 2008 p. 131); he also states that a very low score in men “is suggestive of a failure in the development of normal masculine identity. In women, it suggests insufficient assertiveness for effective adjustment.” (p. 132). Some examples of scale items are: it is difficult to make me cry; it is foolish to waste time thinking about love and romance; large insects and reptiles bother me; I would pick up a non-poisonous snake with my bare hands; crude and vulgar situations displease me. This instrument, however, is widely used in personnel selection, especially in public tenders (Pereira & Bandeira, 2009). Despite the scale’s name revision to “Mental Toughness vs. Sensitivity” in the 1994 version (Comrey, 2008, p. 132), the Brazilian version still considers it an attribute intrinsic to masculinity and femininity.

From the perspective of sexual and gender diversity, both instruments present serious ethical implications, contradicting the CFP’s view, which has been promoting the debate on human rights in general and in the context of sexual and gender diversity. Resolution CFP nº 001/99 (CFP, 1999), in its second article, states that psychologists should contribute “to a reflection on prejudice and the disappearance of discrimination and stigmatization against those who exhibit homoerotic behaviors or practices” (p. 2). To deepen this analysis, this article will proceed in the following steps: initially, the history of psychological assessment of gender and sexuality will be presented. Next, the potential discriminatory biases of the IFP and CPS will be discussed. Finally, concluding remarks on the importance of revising psychological assessment practices in light of human rights and contemporary knowledge will be presented.

## Historical Overview of Gender and Sexuality Assessment in Empirical Psychology

The examination of the supposedly natural difference between men and women is a relatively recent idea. The historian Laqueur (2001) demonstrated that it emerged in Western thought in the 18th century, driven by political interests that found in the study of these differences a justification for maintaining male hierarchy in societal organization. The predominant scientific model until the 18th century was the single-sex model. Originating from Greco-Roman medicine, it understood the anatomical differences perceived in the genitals as different degrees of the same species. The vaginal canal was seen as an inwardly turned penis, which, with the force and intensity of “vital heat,” would evolve to anatomical completeness. The single-sex model would be of a world “where the boundaries between masculine and feminine are of degree and not of kind, and where reproductive organs are but one sign among many of the body’s place…” (Laqueur, 2001, p. 41).

In the model that emerged thereafter, men and women were assumed to have specific and necessary properties. One of the sciences dedicated to studying this model was phrenology, which claimed that the shape of women’s skulls evidenced the development of brain regions necessary for intellectual success, while also showing developed regions related to motherhood (Staum, 2003). Individuals, who until then were evaluated by their religious attitudes or aristocratic values, began to be evaluated by their conformity to their biological and, later, psychological “nature” (Fausto-Sterling, 2008; Foucault, 1998; Katz, 1996). That is, the set of ideas stemming from common-sense psychology used at the time to justify qualities desirable for women and men gained support as these conceptions were incorporated into the sciences consolidating at the time. Scientific psychology emerged at the end of the 19th century, concerned, among other things, with these differences. Examples include research on greater male intellectual variability, on the maternal instinct and its relationship with the so-called feminine nature (Shields, 1982). These issues were examined under the influence of the Darwinian perspective. In “The Descent of Man, and Selection in Relation to Sex” (1871/1933), Darwin offered a hypothesis to explain his observation that, in many species, only males develop a greater variety of secondary sexual characteristics (peacocks’ tails, for example), unlike females. Variability was accepted as a mechanism of the evolutionary process (survival of the fittest and transmission of the most adaptive variations), and it soon became conventional that it was a positive attribute legitimized by evolutionary theory. Thus, greater male variability became a convenient explanation for a large number of differences between women and men at the time, not only in physical but also in mental attributes (Shields, 1975). According to the variability hypothesis, even if more men than women with physical and mental problems were found in asylums of the period, this was compensated by there being more men in society with values considered positive, such as genius and fame (Shields, 1982). One piece of evidence for this hypothesis was provided by studies that compiled illustrious people and traced their genealogy, noting little prevalence of women. Galton (1869), Darwin’s nephew and a precursor to psychological assessment, conducted research in this regard. The idea was that individual attributes, such as greater intelligence and physical strength attributed to men, would explain adaptation to the environment, culminating in personal eminence, these attributes being hereditary. Rare were references that context, not heredity, would explain the low prevalence of women in these studies (Shields, 1975).

With the development of intelligence tests (then called mental tests), this perspective gained momentum. Differences were found in tasks such as idea association, color preference, handwriting, image memorization, reading speed, and the ability to care for young children (Morawski, 1985). In the United States, for example, where psychological research was developing widely, this male-privileged bias took hold strongly (Minton, 2000). Differences between men and women, found in psychological research, automatically converted into expectations regarding social role performance. Thus, the psychology of the time ensured that the hypothesis of less variability served not only as an explanation for women’s disadvantaged social status but also as its justification (Shields, 1975). Stanley Hall (1904), among others, believed, for example, that it was imperative to educate girls separately from boys, so that they would be prepared for motherhood and domestic work, something considered innate to the female condition.

This thinking began to be challenged when the first women entering the field of psychological research published their studies (Minton, 2000). Helen Thompson Woolley reviewed various studies investigating differences between women and men in motor and sensory skills (Thompson, 1903). She concluded that these differences were too small to be considered significant, although some studies favored women. Her conclusion was that “sex differences in psychological terms seem to be largely due not to differences in average capacity, nor in the type of mental activity, but in the social influences exerted on the developing individual from early childhood to adulthood” (p. 182).

However, it was Leta Stetter Hollingworth who conducted research that challenged the prevalent view regarding female variability – and thus inferiority (Silverman, 1989). Questioning one of the central ideas of the time, the author also demonstrated that the population with intellectual disability was similar between women and men. By analyzing the age of residents in New York City institutions, Hollingworth (1914) found that, although there were more boys than girls, the number of incarcerated women increased to match that of men as they aged. For Hollingworth, there were no deterministic explanations for this phenomenon. Her hypothesis was that intellectual disabilities among women were rendered invisible due to their social role. Since mental ability was not expected of women at the time, they were not evaluated early. Therefore, they only entered institutions when, due to age, they were no longer suitable for so-called feminine tasks: “Women have been and are a dependent and non-competitive class, and when they have some diminished capacity they can more easily survive outside institutions, since to maintain themselves in society they do not have to compete mentally with each other, as men do” (p. 515)^1^. Woolley and Hollingworth helped demonstrate that the differences between women and men, crystallized by the psychological thought of the time, were hostage to institutionalized androcentrism, that is, a bias that analyzed research results from a perspective that privileged men, attributing to them socially positive values considered innate. Although their works are recognized today as precursors of feminist psychology, they were largely ignored (Silverman, 1989). Psychology at the time served as a basis for denying women access to various institutions, such as work, higher education, and suffrage. Furthermore, they were classified as maladjusted (hysterics) when they deviated from their expected social role, such as motherhood, domestic work, and sexual modesty (Gilman, 1985).

It should be noted that the difference between men and women was not only evaluated in relation to social adaptation but also sexual adaptation. In 1869, the Hungarian journalist Karol Maria Kertbeny coined the terms homosexual and homosexuality in a text opposing the Prussian penal code, which condemned this type of conduct. Later, the term was appropriated by psychiatrist Krafft-Ebing in his theory of sexual deviations, associating homosexuality with pathology (1886/2011). In Psychopathia Sexualis, the author examined sexual practices from the emerging Darwinian perspective that any sexual behavior not aimed at procreation was maladaptive. Krafft-Ebing was also one of the pioneers in documenting cases of people who wished to live or were already living as a sex different from that assigned at birth. At the time, however, sexual behavior and what is now called gender identity were not considered distinct categories (Drescher, 2010). This was evident both in theories claiming that homosexuals had a “feminine soul” in a male body and in Freud’s hypothesis of “inverted” sexual development (Freud, 1920/2011), or in the generic notion of sexual inverts propagated by Krafft-Ebbing and sexologist Havelock Ellis (1927/2013). Magnus Hirschfeld is credited as the precursor, between 1910 and 1920, of the distinction between same-sex desire and the desire to live as someone of a different sex from that assigned at birth, which he called transvestism (Drescher et al., 2012). However, as we will see below, such distinctions were only widely accepted later, thanks to the work of Henry Benjamin and John Money. Thus, based on the theories of the time, until the second half of the 20th century, psychology and psychiatry professionals offered all kinds of corrective therapies for sexual and gender variations (Drescher, 2010).

With the popularization of psychological assessment in the early 20th century, numerous instruments were developed to measure a vast range of aptitudes: verbal, mathematical, among others. Research on the differences between men and women also increased, but was shrouded in great confusion, since, in general, studies did not reach a conclusion that clearly favored one or the other (Morawski, 1985). It was Lewis Terman and Catherine Miles who, in 1936, offered a solution to this impasse. Analyzing the various tests available at the time, they selected only the items in which men and women differed, to form a new instrument capable of analyzing not the difference between men and women in a given test, but the differences between “masculinity” and “femininity” in the response pattern. That is, a measure of “psychological sex,” where the differences between men and women found in previous psychological research would establish what was taken as the standard of femininity and masculinity. To reduce the possibility of influence on the response, the instrument was generically named the Attitude Interest Analysis Survey (AIAS; Terman & Miles, 1936).

In this instrument, masculinity and femininity were understood as a bipolar and unifactorial construct. This means that masculinity and femininity were opposite poles of a single continuum; in the case of the Terman and Miles test (1936), this amounts to different responses across the 456 items of word association, attitudes towards feelings, interests, and opinions divided into seven subscales. The femininity score was marked by negative responses to questions such as: liking to ride a bicycle, playing with snakes, seeing a flower or a star in an inkblot, and the masculinity score, in turn, by responses such as: claiming to see a bat in the same inkblot or disliking foreigners, intelligent women, and dancing. It is notable that the test’s definition of femininity is hostage to the androcentric view that came from the first studies in this area: “Submission, docility, inferior constancy of purpose and a general lack of aggressiveness reflect their weaker conative tendencies” (Terman & Miles, 1936, p. 2).

In the wake of Terman and Miles’s original proposal, many tests were created to exclusively assess masculinity and femininity, or to assess this factor within a larger set of characteristics. One such scale is the Guilford-Zimmerman Temperament Survey (Guilford & Zimmerman, 1949), a personality test that includes a masculinity and femininity scale, which served as inspiration for the M scale of the CPS (Comrey, 2008). Another example is the Minnesota Multiphasic Personality Inventory (MMPI) (Hathaway & McKinley, 1940). This instrument was developed for the clinical psychology context, with the aim of measuring traits associated with psychopathology and psychological disorders. The MMPI has a masculinity and femininity scale inspired by the Terman and Miles measure; however, with most items developed specifically for this instrument. However, instead of choosing items that distinguish men from women, like the Terman and Miles scale, the test especially uses items that distinguished homosexuals from heterosexuals (Shields & Dicicco, 2011). In the North American context, psychoanalytic theories gained momentum, and with them, the understanding that sexual difference was a central psychic attribute for personality, not accessible to consciousness (which justified an external evaluation), and that it would follow a development considered normal (Moranski, 1985). That is, an emotionally healthy person would be someone who was assigned female sex at birth, identified as a woman, conformed to and behaved according to the social expectations appropriate for the female personality of the time, and was heterosexual. At the time’s assumption, “feminine” men and “masculine” women were automatically considered sexual inverts. Therefore, this type of gender assessment aimed to evaluate these supposed incongruities (Shields & Dicicco, 2011).

The 6th edition of the International Classification of Diseases (ICD), published in 1948, was the first publication by the World Health Organization to include a classification of mental illnesses. Following the trends of the time, gender and sexuality variations (homosexuality and transvestism) were considered similar phenomena and appeared in both ICD-6 and the subsequent 1952 edition (ICD-7) as inclusion terms for the diagnosis of sexual deviations, which were classified as integral to a pathological personality (Drescher et al., 2012). The American Psychiatric Association, following the same trends, published, in 1952, the first edition of its manual (DSM-I), listing everything that psychiatry considered as mental illness at the time. In this manual, sexual deviations, including homosexuality and transvestism, were also classified as personality disorders. Techniques, which we can now consider as torture, were used to alter sexual behavior (Gilman, 1985). During this period, instruments developed for the diagnosis of homosexuality abounded. Among them, the diagnostic indicators of homosexuality in human figure drawing (Gardner, 1969) and the interpretation of the Rorschach test according to Schaffer (Andersen & Seitz, 1969). These tests sought clinical differences between heterosexuals and homosexuals, differences that, when found, justified psychological deficits of homosexuals in relation to heterosexuals, contributing to the maintenance of the pathology status. These works did not take into account, for example, that such problems could be the result of the great social segregation and stigma experienced by this group.

However, another trend in the study of sexual and gender variations began to question the prevalent view. Studies such as that of anthropologist Margaret Mead (1935/2011), which showed that men and women have completely distinct social and sexual roles in other cultures, gained influence at this time. Also from this period are the works of Alfred Kinsey and colleagues, who, in 1948, published a book on the sexual behavior of American men, finding that homosexual experiences were relatively common in the United States. Works by Hooker (1957, 1958) are from the same period, who conducted the first study comparing non-clinical samples of homosexuals and heterosexuals. Hooker concluded that homosexuality did not constitute a clinical entity and should not be associated with psychopathology. Furthermore, this author’s work questioned the use of psychological assessment to identify sexual orientation, stating that “its doubtful validity makes its value questionable” (Hooker, 1958, p. 51).

At the same time, John Money published his studies on children born with intersex conditions (Money et al., 1955, 1957). Analyzing cases of medical sex assignment procedures in children born with ambiguous genitalia, Money believed that parental attitudes would have a strong effect on the child’s acceptance of the clinically assigned category. Money was a pioneer in distinguishing between sex and gender, theorizing that the sense of being male or female was acquired primarily through environmental factors. For Money, there was a difference between (a) anatomical and physiological factors, i.e., chromosomal, hormonal “sex,” gonads, and external and internal genitalia; (b) gender socialization in early childhood; and (c) psychological characteristics - gender social role - acquired through this socialization. Later, Money differentiated gender identity - the private sense - from gender social role - the public expression of gender identity (Money & Ehrhardt, 1972).

In the mid-1960s, in the wake of Money’s work, the first clinics emerged offering genital modification procedures for adults seeking such treatment. Although experiments with these surgeries began in the 1920 s and 1930 s, based on the idea that gender was fixed early and that efforts to change it were fruitless, medical body transformation techniques proved to be an acceptable therapeutic alternative. Benjamin (1966) is credited with popularizing the term transsexual and raising awareness about the need for health care for trans people, while psychiatry and medicine at the time considered them confused homosexuals, inverts, and schizophrenics (Drescher, 2010). For Benjamin, for example, a transsexual woman would be a person of female gender “trapped” in a male body, with the only therapeutic alternative being hormonal and surgical treatment aiming to perform the “transition” to the “other sex” (male to female - or female to male). Benjamin inaugurated the distinction between “transvestism” and transsexuality, which had been mixed until then, showing that in the former case, the desire for bodily change and identity affirmation was not at stake. Although some of these notions have recently changed, as we will see below, thanks to Benjamin, the idea that trans people should not be subjected to conversion therapies was scientifically affirmed, consolidating the therapeutic approach used to this day (Coleman et al., 2012).

The concept of gender had a great impact on how biological and psychological determinism was conceived, driving a new wave of feminism, including in psychology (e.g., Unger, 1979). The idea that sex would be biological and gender social helped to confront the beliefs that there would be, in this sphere, an equivalence between nature and culture. In other words, the understanding solidified that a large part of the differences attributed to men and women was due to socialization. In other words, people were taught to have masculine and feminine characteristics and to identify as men and women. Previous psychological research began to be understood as studies on gender, a category created in reference to biological bodies, but not determined by them. These theoretical and political transformations can be seen in the medical manuals of the time. The idea that variations in sexuality and gender did not constitute a personality deviation appeared for the first time in ICD-8 (1965), in separate diagnoses - homosexuality and transvestism - without presenting definitions. DSM-II was published in 1968 and maintained the same separation and nomenclature. To ICD-9, in 1975, the category transsexualism was added, to account for the new treatment strategies presented in previous decades. Homosexuality still appeared as a disorder in ICD-9; however, influenced mainly by political activism, the seventh impression of DSM-II, in 1974, no longer listed homosexuality as a disorder category. In this manual, the previous diagnosis was replaced by “sexual orientation disturbance,” diagnosing homosexuality as a disease only in cases where the individual feels discomfort with it and wants to change (Drescher, 2010). DSM-III, published in 1980, maintained the category of ego-dystonic sexual orientation to account for the same cases. Based on the understanding that this category only served to legitimize ineffective conversion therapies, this diagnosis was removed from the revised version of DSM-III in 1987 (Drescher, 2010). ICD-10 of 1990, the most current edition, includes the diagnosis of ego-dystonic sexual orientation. However, ICD-11 removed this category, completely depathologizing homosexuality (Cochran, 2014).

These new views also serve as a trigger for a series of movements in civil society and in psychology societies, moving away from a model that previously sought to justify differences from an exclusionary bias, towards research that seeks, for example, the genesis of gender violence, sexism (Eagly et al., 2012). Similarly to what happened with sexism, when homosexuality itself ceased to be a psychological problem, attention in this field turned to those who considered it a deviation. George Weinberg published, in 1972, Society and the Healthy Homosexual, popularizing the term homophobia. Sexism and homophobia became consolidated as topics of academic analysis and intervention and banners of the feminist and homosexual movements. Research that, until then, had been focused on curing and diagnosing homosexuality, began to seek the roots of prejudice and strategies to revise discriminatory psychological practices, including psychological assessment (Snyder, 2011). Since then, psychology has been dedicated to reversing the damage caused by the stigma it had helped to build. In 1975, the American Psychological Association published a document stating that homosexuality itself does not imply impairment and further appealed for “all health professionals to take the initiative to remove the mental illness stigma that has long been associated with homosexual orientations” (Conger, 1975, p. 633).

Although inconsistencies were occasionally found, the masculinity and femininity assessment model proposed by Terman and Miles (1936) remained a reference for more than three decades. This technique suffered its greatest criticisms only in 1973, with Anne Constantinople’s work. This researcher was the first to gather evidence that this type of assessment lacked theoretical support, attacking the idea that the assessed traits would be long-lasting, related to anatomical differences, primary experiences, and would serve to distinguish men from women in terms of attitudes and behaviors. According to Constantinople (1973), the fact that existing studies showed that men and women did not respond as expected to these scales should not serve to label them as deviant, inverted, or in conflict, but to highlight the problems of the construct in question. Constantinople suggested that there was no single bipolar dimension (which would involve masculinity at one extreme and femininity at the other), which was not affected by development, nor would it be related to sociodemographic markers. On the contrary, many men and women equally presented characteristics associated with masculinity and femininity, characteristics that normally vary throughout the life cycle and according to social class and culture. Furthermore, the literature did not support the idea that homosexual men would have a psychic functioning equivalent to feminine women and lesbian women to masculine men. Thus, the idea of sexual inversion should also be revised. For Constantinople, these scales measured “the test-taker’s expectation of how they should answer questions like ‘I would like to drive a race car.’ This response is based on the stereotype of the woman’s role derived from common sense and data that are probably 20 years older than the person taking the test” (Constantinople, 1973, p. 403). Faced with the problems of this type of measure, Constantinople asked: “if M-F scales reflect a number of traits such as aggressiveness, sensitivity, self-confidence, etc., is there any gain in combining these measures from what would be most characteristic of men and women?” (p. 405). That was not exactly what happened.

In 1974, Sandra Bem introduced the Bem Sex Role Inventory (BSRI) as a new way to measure masculinity and femininity. The BSRI was constructed at the time of the emergence of cognitive psychology and information processing theories, based on the idea of sex-typing, that is, assuming that people internalize socially desirable norms and behaviors for men and women and begin to operate from them (Bem, 1974). In the BSRI, the respondent is asked to describe themselves on 60 personality characteristics, on a 7-point Likert scale (1 = disagree, 7 = agree). These characteristics were classified as masculine, feminine, and neutral based on what was most desirable in North American society at the time (Bem, 1974). Among the feminine characteristics are, for example, compassion, love of children, and tenderness; among the masculine, assertiveness, strong personality, and leadership ability; and among those considered neutral, being adaptable and conventional. The novelty introduced by Bem concerns the way the test is evaluated. A person with high rates in both dimensions (masculinity and femininity) was classified as “androgynous”; low in both, “undifferentiated”; and high in one dimension but low in another, sex-typed as “masculine” or “feminine” (Bem, 1977). Bem’s measure no longer prescribed the correspondence between “biological sex” and characteristics attributed to men and women, completely ending the idea of deviation and sexual inversion. In Bem’s model, in fact, the best-adjusted people would be those with equivalent values of masculinity and femininity (androgyny hypothesis). These “balanced” individuals would not be sex-typed and, therefore, would be more flexible in their concepts and behaviors, and psychologically healthier (Bem, 1974).

Although the implications of Bem’s scale were liberating for the time, especially in relation to the idea of androgyny, several criticisms emerged regarding this approach. One of them came from research dedicated to studying socialization, pioneered by Eagly (1987). This theoretical perspective seeks to understand how society conceived, for example, that women should have compassion and men, leadership ability, and how people would be taught to act according to these characteristics. For Eagly, both men and women can have “compassion” or “leadership ability,” as long as they have the social support that allows them to exercise these capacities. What this theory points out is that there is societal pressure, according to each culture and historical context, unequally dividing what is expected for men and women and making them adapt to this division throughout their development. From this model, many experiments showed how young children are “taught” to be boys and girls, often in subtle ways. In a classic study, mothers were asked to interact with infants under one year old. If the baby was called a girl, a doll was more frequently offered. If the same baby was called a boy, a train was more frequently offered (Will et al., 1976). In another experiment, Condry and Condry (1976) showed university students a video of a baby’s reactions to receiving a toy. If they were told the baby was a boy, students more often classified its behavior as anger. On the other hand, if they were told the same baby was a girl, students classified the same behavior as fear. Other studies also show how parents react negatively when their male children play with dolls or dress in girl’s clothes, and vice versa (Langlois & Downs, 1980).

In principle, social role theory does not invalidate Bem’s position that people would identify with the gender roles available in a given culture and begin to act from them. However, this theory points out that characteristics are not attributed to men and women randomly, as there is a social dynamic that promotes difference in this attribution. The conclusion regarding socialization research is that, when the distribution of roles becomes more egalitarian, much of the difference detected between men and women will disappear (Eagly, 1987). In this way, social role theory recognizes psychology as one of the institutions that promotes the maintenance of gender roles, and advocates that itworks towards correcting inequalities, and not the opposite. The use of the BSRI, in this sense, even if it reflects characteristics of men and women at a given time, reinforces, for example, that it is masculine, and not feminine, to have a strong personality, naturalizing gender inequality.

Another critical perspective concerning Bem’s proposal is that of Janet Spence (for a review: Spence, 2011). Spence’s proposal states that gender is more than adherence to social roles. Since the mid-1980s, Spence suggested that, instead of characteristics and behaviors typically associated with men and women, masculinity and femininity should be conceptualized as gender identity. For Spence, most people are confident about their gender identity, and this identity remains secure even when it does not conform to accepted standards. For example, a person can have a clear female gender identity, despite the fact that they are not a mother, if that constitutes their particular definition of what it is to be a woman. Spence conducted a series of experiments to support her hypothesis (Spence, 1993; Spence & Buckner, 2000). In these experiments, she asked people to rate on two five-point scales how masculine and how feminine they thought they were. She then calculated the correlation between this self-assessment and external measures of gender, such as the BSRI. The self-assessment of femininity was high and that of masculinity was low in people who identified as women, and the opposite occurred in people identified as men. Furthermore, almost all correlations between self-assessment and other measures were not significant. “Even if it is not articulated, the constancy that people feel in relation to their gender identity can give rise to the illusion that all observable differences in the characteristics and behaviors of men and women contribute to an underlying psychological property, masculinity-femininity, which could be assessed from its supposed manifestations” (Spence, 1993, p. 634). The result of these studies, therefore, supports the hypothesis that the most important thing in gender assessment is self-designation of identity. In other words, people are not mistaken when they say they are men or women, even if psychology and its assessments say otherwise.

Finally, the most forceful critique of the BSRI model of assessment seems to have come from the debate regarding trans people. Healthcare for these individuals through body modification procedures, inaugurated by Henry Benjamin and crystallized in ICD-9, became even more institutionalized with the publication of DSM-III in 1980, where transsexualism appeared again as a diagnostic category. DSM-III also marked the feminist movement’s victory with the removal of the “hysteria” category, which proved clinically irrelevant and discriminatory (Ussher, 2013). With the publication of ICD-10 in 1990 and DSM-IV in 1994, transsexualism was modified to gender identity disorder. This change reinforced Benjamin and Money’s idea that trans people suffer from having something like a gender “soul” trapped in another body. At this point, despite the ongoing debate, the institutionalization of the diagnostic criteria for transsexualism and, later, gender identity disorder, was made available both for trans people—since they could more easily access body modification procedures—and for clinical medicine, which would not need to perform them experimentally or clandestinely (Drescher, 2010).

The study of the BSRI with trans people revealed interesting data evidencing the existence of other forms of gender construction. For example, studies revealed that trans women did not identify completely with the characteristics associated with cis women (the same for trans and cis men), differing even among themselves (Fleming et al., 1980; Herman-Jeglińska et al., 2002). Furthermore, the same research began to show that some trans people did not seek body modification procedures out of rejection of masculinity or femininity, and others simply did not seek them. In other words, they reinforced Spence’s point of view: for some trans people, gender identity was independent of both sex assigned at birth and what was socially expected for men and women. And in this case, the BSRI failed doubly: first by privileging external assessment over gender identity, and, second, by operating through a binary model (masculine/male and/or feminine/female), when evidence suggests other possibilities.

Muehlenhard and Peterson (2011) reported an exemplary anecdotal case in their article. In a dissertation defense, a student who had compared men and women was questioned by two professors. One of them asked: “Did you assess the chromosomes, ask about the genitals?” With the student’s negative answer, the professor stated: “Then you were researching gender differences, not sex differences.” The other committee member asked: “Did you measure masculinity and femininity? Did you use the BSRI?” The student again said no. “Then you investigated sex differences, not gender differences!” Muehlenhard and Peterson’s case reveals how much psychology was still tied to the model of difference between sex (biological) and gender (social) in terms of the male/female binary, a model also present in the clinical management of trans people, as health professionals did not encourage them to live openly as “trans,” but to make a complete transition to the “opposite sex” (Drescher, 2010).

This model began to be questioned with the emergence of studies showing that different gender constructions are present in other cultural and historical contexts, including the figure of a “third sex” (Herdt, 1996). In addition, the idea gained strength that gender and sexuality are different arenas of human experience, and also of political activism. In the 1990 s, the transgender category emerged in the North American context, encompassing the set of gender variations that includes trans people, crossdressers, drag queens and drag kings, and anyone who transgresses the binary gender model, even if they are not willing to undergo body modification procedures (Davidson, 2007). Thus, many trans people began to “come out” to the public scene, reinterpreting their experience from the transgender category (Valentine, 2007). It should be noted that there is no consensus on the use of the term transgender in Brazil. Currently, gender variations are usually referred to using the categories travesti and transsexual, or, more recently, trans people (Carvalho & Carrara, 2013).

Of great influence on this movement were the points made by Suzanne Kessler (Kessler & McKeena, 1978; Kessler, 1990, 1998). Observing the clinical management of intersex children, Kessler questioned John Money’s position that sex assignment would definitively influence future gender identity. Her justification came from the case of some adult intersex individuals in whom gender identity did not agree with the sex assigned at birth by medical professionals and reinforced by the family through the children’s upbringing. Money himself proved mistaken when, years later, the questionable ethical treatment given to his patients and the way he conducted his research to mask results that contradicted his point of view came to light. See: Diamond and Sigmundson (1997) and Colapinto (2001). This medical management of intersex cases would perpetuate the idea that gender authenticity resides in “sex nature,” and not in its assignment, whether by doctors, family, or the individual themselves. For Kessler, we make a gender attribution every time we meet a person, and this is independent of biological characteristics. For example, “if a trans woman is assigned the designation ‘man,’ it is being stated that she has not yet transitioned or has not done it properly. On the other hand, if the designation ‘woman’ is attributed to this person, it means that, for all intents and purposes, she is a credible woman” (Kessler, 1978, p. 14). Kessler proposes that genital ambiguity in intersex children was not accepted as an option not because it was threatening the child’s life (these cases are rarely medical emergencies), but because it threatened their culture. Medical procedures would guarantee the assignment of only one of the two culturally accepted sexes (male - penis, female - vagina). Sex and gender would therefore not be distinct categories, since the binary social notion of gender is implicit in the attempt to find the “true sex” in chromosomes, gonads, and external and internal genitalia. “The non-normative is converted into normative, and the normative state is considered natural. Genital ambiguity is remedied to obey a ‘natural,’ that is, culturally unquestionable gender dichotomy” (Kessler, 1990, p. 25).

When the distinction between sex and gender becomes less important, people who do not fit into the categories assigned at birth are no longer treated as individuals who supposedly were born with a defect; that is, the problem is also referred to society’s limited categories (Kessler, 1998). In other words, trans people (and also intersex people) would not be sick individuals whose only option would be medical repair, but rather emotionally healthy individuals whose gender expression was limited by social expectations causing suffering. Thus, the model of transsexuality propagated by Benjamin also loses strength. There are people who have always considered themselves women, even though they were born with a penis, just as there are people who have always considered themselves men, even though they were born with a vulva. In the new conception, these people do not perform a “transition” to the “other sex,” but rather adapt their body and civil records to their gender identity, regardless of the sex assigned at birth. The transgender category emerges as an alternative to the old medical model, in an attempt to affirm gender variations as variations of normality. This new view on gender unites the banners of the social movement, expanding the focus of the struggle for gender equality between women and men, towards equality between cis and trans people. The movement of gays, lesbians, and bisexuals (LGB) begins to include the demands for civil rights of trans and intersex people (LGBTI), and a part of the feminist movement that was increasingly concerned with intersectionalities such as social class, race/color/ethnicity, also begins to embrace the trans agenda, towards transfeminism (e.g., Serano, 2007)^2^.

Many areas of knowledge are beginning to reform their theories and practices from this point onwards. It would be no different with psychology, which helped create a model based on the pathologization of gender variations, focusing on what “went wrong” and therefore should be corrected, moving towards a positive affirmation model, focusing on the stigma associated with gender variations and the resulting health disparities (Bockting, 2009). Reflections of these changes are found in the publication of DSM-5, which depathologizes gender identity and now only classifies gender dysphoria (discomfort) (Cohen-Kettenis & Pfäfflin, 2010), and ICD-11, which removed this condition from the list of mental disorders (Drescher et al., 2012). Although there is room for progress, such changes propose reducing the stigma associated with the diagnosis of mental illness, without prejudice to access to body modification procedures for trans people who need them (Drescher, 2014).

The effort of contemporary research has been to broaden the concept of gender, so that it accounts for the experience of both cisgender and transgender people, without exoticism or exclusion; for example, recognizing that it is not only trans people who modify their bodies to affirm their gender: cisgender women and men use silicone prostheses, exercise, and use hormones to affirm their gender. Tate et al. (2014) listed five categories that cover the ideas discussed in this article: (1) sex assigned at birth (also called sex designation); (2) gender identity, or self-designation as a man, woman, travesti, neutral, queer, or even genderless; (3) adherence to culturally associated gender stereotypes; (4) public gender expressions or performances, through the use of proper name, body language, and clothing; and (5) attitude towards gender, which can be favorable or not (sexism and transphobia; see also: Hill & Willoughby, 2005). This division proves quite useful from a theoretical point of view. From a practical point of view, however, resolving the impasse of the dissertation presented by Muehlenhard and Peterson (2011), two questions suffice, based on self-designation: “How were you assigned at birth?” and “How do you currently identify?”

Additionally, contemporary ideas regarding sexuality should be added to these categories (for a current review see: Diamond, 2009). Today, the concept of sexual orientation has been used to designate sexual desire, regardless of whether it manifests in behavior. For example, a woman may have a homosexual orientation and never have actually been in a relationship with another woman. Sexual identity refers to self-designation as lesbian/gay/bisexual/heterosexual. As with sexual orientation, identities do not always correspond to behavioral patterns. A man may consider himself heterosexual and have sexual relations with other men. This is the case that led to the emergence of the category “men who have sex with men” (MSM). Furthermore, some people may reject traditional identity labels in favor of others such as queer, pansexual, asexual, etc. Finally, positive or negative attitudes towards sexual diversity (homo-, bi-, and lesbophobia) are added to these categories; these do not correspond to stable nosographic concepts, being determined or informed by social dynamics. For example, some people may be born with a penis, have a female gender identity, and, in some cases, be attracted to men, and, in others, to women; an attraction that may vary at different times of life.

## Discriminatory Biases in Psychological Assessment

Chernin et al. (1997) reviewed a series of widely used tests in the United States, including the Beck Depression Inventory (Beck et al., 1961) and the Minnesota Multiphasic Personality Inventory - MMPI (Hathaway, & McKinley, 1940). The authors suggest three biases that should be considered when evaluating instruments with regard to sexual and gender diversity: omission, connotation, and contiguity. The bias of omission occurs when the language used by the instrument ignores the possibility that the respondent belongs to a minority group. The second type of bias is connotation, which occurs when words with negative connotations are associated with minority groups. For example, words like homosexuality appear together with terms like alcoholic, fetishist, maladjusted, suggesting a negative categorization of homosexuality. The third bias is contiguity, which happens when scales aimed at evaluating psychopathology appear together with scales whose purpose is to characterize minority groups.

The analysis of the IFP reveals some biases according to Chernin et al. (1997). Regarding the items that comprise the instrument, examples of omission bias can be found. In the heterosexuality scale (HT), items such as “I like to kiss attractive people of the opposite sex” appear alongside “I like to get sexually aroused” (Pasquali et al., 1997, p. 26). This suggests that the test aims to evaluate heterosexual sexual interest, leaving homosexual sexual interest omitted from the instrument, since it is not possible, at the same time, to be attracted to same-sex individuals and interested in sexual arousal. The HT scale was originally created to assess “the desire to maintain relationships, from romantic to sexual, with individuals of the opposite sex. The subject with a high score on this factor is fascinated by sex and related topics” (p. 39); however, in the instrument’s manual, a young person with extremely low scores on this scale is interpreted as follows: “for a young person of such an age (22 years) interest in sex seems abnormally absent (could it be repression?)” (p. 57). The test does not assume that the young person in question may be homosexual; instead, it interprets the score as repressed heterosexuality, that is, negatively, constituting a clear connotation bias. Finally, the mere presence of the HT scale in the test constitutes a contiguity bias, since the IFP is widely used in subclinical contexts (Peres & Santos, 2006; Irigaray & Schneider, 2007), where homosexuality is implicitly associated with psychopathology. It is noteworthy that even the technical criteria point to problems with the HT scale. When the test is analyzed for its factorial structure, the needs associate with each other, except for heterosexuality (Pasquali et al., 1997), which questions the relevance of this scale in relation to the other dimensions of the evaluated construct. The same occurs in a recent review of the IFP’s factorial structure, which pointed out that “the unidimensionality of the factors can be questioned, which suggests, at least, that the subjects perceived distinct contents in relation to the items that form each factor” (Araújo, 2004, p. 9).

Applying Chernin et al. (1997)’s criteria to the Comrey Personality Scales (CPS) reveals discriminatory biases similar to those found in the IFP. Omission bias manifests in the CPS’s Masculinity and Femininity (M) subscale, where items presuppose a binary and stereotypical gender pattern, omitting the diversity of gender experiences and identities. Connotation bias is evident in the description of individuals with extreme scores on the M scale, associating characteristics like “strong,” “stubborn,” and “tough” with masculinity, while “cry easily” and “are bothered by the sight of insects” are linked to femininity, carrying implicit value judgments. Contiguity bias is also present, as the M scale, alongside others that assess personality traits, can lead to the interpretation of gender variations as “pathological social conduct impairments,” reinforcing stigmas and prejudices. Finally, it exposes a portion of the trans population to unnecessary embarrassment, since there is no other option than to identify with the instrument’s (binary) theoretical model.

The question remains as to how these tests could have been approved for use by the official system of the Federal Council of Psychology in Brazil, SATEPSI, despite such blatant biases and given the history of revision of psychological theories in the context of sexuality and gender. The fact that these instruments remained valid and available for use until 2018 is indicative of the operational mechanics of SATEPSI during that period. The system’s approval criteria focused heavily on psychometric properties—validity and reliability evidence—rather than a critical content review regarding human rights. It is important to clarify that their removal in 2018 was not necessarily due to a specific intervention regarding their discriminatory content, but rather due to the expiration of the validity of their normative studies (which typically have a 15-year limit in the SATEPSI system) or the failure to present updated studies. This phenomenon was not exclusive to the IFP and CPS; other widely used instruments in Brazil,,also faced challenges or required updates to align their theoretical constructs—often imported or dated—with contemporary ethical demands. However, the IFP and CPS are highlighted here because their specific scales for sexuality and gender provide the most direct evidence of the disconnect between the CFP’s political resolutions on human rights and its technical protocols for test approval.

Since the 1970 s, with the publication of the first professional code of ethics, the Federal Council of Psychology (CFP) has paid attention to the theme of human rights. This discussion strengthened during the 1990 s with the creation of the CFP’s National Human Rights Commission and, later, with the mandatory establishment of human rights commissions in regional councils. The various re-editions of the code of ethics attest to this trend, and in its last revision, the very first fundamental principle states that “the psychologist shall base their work on respect for and promotion of freedom, dignity, equality, and the integration of the human being, supported by the values that underpin the Universal Declaration of Human Rights” (CFP, 2005, p. 7).

Although a more generic concern with human rights was present to some degree in CFP regulations, its relationship with psychological assessment is recent. Only in 2010 did texts clearly discussing this relationship emerge, especially with the publications: Psychological assessment: guidelines in the regulation of the profession (2010), Year of Psychological Assessment – Generating Texts (2011) and the Report of the Thematic Year of Psychological Assessment (2013). Two recurring arguments in these texts are, firstly, the recognition that psychological tests were created from an adaptationist perspective and that “the act of assessing implies the emission of judgments and values” (Anache, 2011, p. 17). Secondly, the idea that, to comply with human rights, it is necessary to “pay attention to the technical characteristics of the instruments (such as different evidence of validity and reliability)” (Reppold, 2011, p. 24). This refers to the fact that, historically, psychological assessment fosters social exclusion practices, especially given that assessment “was reduced to the administration of isolated tests without considering the context of application, nor even the need to adapt the instruments to local norms” (Reppold, 2011, p. 23). This type of practice is still present, since most ethical infractions reported to the Federal Council of Psychology concern complaints regarding psychological assessment. Complaints such as: extrapolation of the use of tests to situations different from those for which they were designed; false or limited statements based on the tests; and lack of confidentiality (Anache & Reppold, 2010). These points of view seem to support, on the one hand, the belief that by establishing minimum psychometric criteria for the validity and reliability of instruments, ethical criteria will also be guaranteed, that is, the belief in the ambivalence between technical and ethical criteria. On the other hand, they adopt a very broad view of human rights, which leads to a generic notion of violation of these rights, thus losing the specificity of the violated rights in question. To avoid the risks of ineffectiveness in applying the human rights principles present in these viewpoints, it is necessary both to establish how the ethical quality of instruments can be guaranteed (whether solely through scientific responsibility or not) and to distinguish, among human rights, the fundamental ones, in order to identify which ethical perspective is sought.

It is clear that the implementation of SATEPSI in 2001 changed the landscape of misuse of psychological assessment techniques in Brazil. Before the system was established, as Hutz (2009) recalls, tests commonly used in Brazil utilized data from economically developed countries. The norms related to these countries, among others, led to an assessment not consistent with individuals from other social groups different from the instruments’ origin context. The control exercised by the CFP through SATEPSI raised the quality of tests available in the country and the performance of psychologists in this area. On the other hand, although the resolution regulating SATEPSI states that periodic revision of the conditions of methods and techniques used in psychological assessment is necessary “with the aim of guaranteeing services with technical and ethical quality to the users of these services” (CFP, 2003, p. 1), there is no mention, neither in CFP Resolution nº 002/2003 nor in CFP Resolution nº 006/2004 (CFP, 2004) that amends it, of the procedures to be adopted to ensure this ethical quality. Furthermore, there is no single mention in these resolutions regarding human rights and their principles, with the resolutions being restricted to technical criteria. It is reinforced that at the same time the pioneering CFP resolution, 001 of 1999 (CFP, 1999), which prohibited reparative therapies from homo to heterosexual, and manifestations of prejudice against sexual diversity, had already been published. Again, a paradox.

Our hypothesis relates to the fact that in the 1970 s, a division occurred in Brazilian psychology between positivist psychology, focused on empirical research, and critical psychology, more linked to social sciences (Jacques et al., 2014). Simultaneously, the interdisciplinary field of gender and sexuality studies developed in Brazil (Borges, 2014; Nuremberg et al., 2011). Despite the growing number of studies in gender relations, especially in social psychology, Brazil still does not have an institutionalized field of study like North American feminist psychology (Nuremberg et al., 2011). Feminist contributions remain limited in Brazilian empirical psychology, where gender is rarely used as an analytical category (Narvaz & Koller, 2007). Although already in 1991 the APA published a document guiding the review of psychological practices taking into account prejudiced biases (APA, 1991) and due to the abject and pathologized visibility to which the LGBT + population was subjected, the validity of several instruments began to be questioned in view of these populations. This was not the case in Brazil, as we will show, a comprehensive review of psychological theories and practices, including gender psychological assessment, taking into account these social markers, has not yet been fully carried out in the country. Therefore, the persistent use of tests like the CPS in Brazil seems to be related to the fact that, unlike the North American context, where these instruments originated, feminist studies in social psychology and psychological assessment have consolidated with few interfaces and with distinct epistemological bases. Thus, areas of Brazilian psychology such as psychological assessment resist the modification of the binary classificatory pattern.

### Final Considerations

The objective of this article was to present the various ways in which psychology has dealt with gender assessment, in order to historically contextualize the IFP and CPS instruments. This extensive historical trajectory, from the single-sex model to the pathologization of gender variance, is what provided the epistemological ground for instruments like the CPS and IFP to be constructed. Without understanding this deep historical root, it is impossible to comprehend how such scales were not only created but accepted as scientific truths for decades. Recognizing the influence of distortions caused by society’s prejudiced expectations, psychology moves from a model that considered differences between men and women natural to one that recognizes gender within a complex system of power relations. In other words, feminist psychology helped add a social dimension to the deterministic model of sexual and gender differences, frequently used as an argument against equal rights, showing that sex and gender are not natural and universal phenomena, but institutional and historical. Isonomy, that is, equality between men and women, is also an institutional and historical phenomenon. However, in the model of isonomy, what is at stake is not the imposition of homogeneity (sexual and gender), but the recognition of stigmatized groups (including by theories that psychology helped to propagate), and who are, therefore, worthy of protection. For example, the IFP is based on Murray’s (1938) theory of basic psychological needs, and it is evident that we have better notions of personality today than we had at the time the theory underlying the instrument was created. Revisions of the theories underlying the tests are necessary, but it is necessary to go further. Psychological practice has been constituted as a tool of adaptation and adjustment by not sufficiently questioning its techniques, which are historically dated, thus instituting “models of being and being in the world according to standards of normality produced as unique and true, inferiorizing and disqualifying the places occupied by the so-called different, abnormal, dangerous” (Bicalho, 2011, p. 90).

For ethical quality principles to be contemplated in psychological assessment, in accordance with human rights, these differences need to be contemplated, not excluded. In the examples cited, it is clear how an instrument can be in perfect harmony with established technical criteria and with the regulatory system, i.e., be valid in the opinion of professionals in the area, and even so, be in disagreement with ethical criteria. The discussion focused on the issue of sexual diversity, but the same may be occurring in other themes involving social markers, such as those related to class, age, schooling, race/color, and religion, among others. With the availability of updated knowledge, it does not seem acceptable, for example, to subject the population to medical procedures, such as surgery, using outdated techniques. In psychology, it is not just about the use of an antiquated measure (the CPS is based on a theory from 1948!); it is more serious, because the categories used to describe subjects have implications for how they construct themselves as such. The type of CPS assessment does not take into account self-designation and does not contribute to the construction of equality between men and women, trans and cis; on the contrary, it is a dangerous strategy that restricts the autonomy of subjects and reinforces archaic stereotypes. Therefore, it is fundamental that psychological practices, with special emphasis on gender assessment measures, be revised in light of the contemporary knowledge presented so far.

In Brazil, psychology has advanced in the recognition and promotion of human rights in its relation to psychological assessment with the publication of CFP Resolution 005 of 2012 (CFP, 2012). Additionally, after the publication of the two original articles that presented criticisms of the instruments (Costa & Nardi, 2013; Costa et al., 2017), two other resolutions (CFP 2018 and CFP, 2022) were published that regulate the use of tests in Brazil, and they clearly incorporate ethical criteria for guaranteeing human rights in the authorization for the use of tests in Brazil. However, many challenges still need to be faced. North American psychology is an example of how an institution that played a central role in legitimizing a stigma was able to recognize its complicity in this process and work to undo its negative effects. This article points out the problem of discriminatory biases present in psychological assessment, long studied in the US context. In a country characterized by such marked social, cultural, and economic differences as Brazil, the work of reviewing psychological techniques must be constant, and professionals trained in the area need to be able to take such a review into consideration.

A direction for such a review can be extracted from the article by Herek et al. (1991). Based on the authors’ positions, some questions can be observed. Does this instrument ignore or deny the existence of any social group? Does this instrument stigmatize any social group? Does this instrument reflect stereotypes about these groups? Does this instrument implicitly suggest that the evaluated characteristics are caused by some intrinsic attribute of these groups? Do the items contemplate diversity? As for the sample: Is the sample representative? Does it include enough diversity to allow for accurate interpretation? As for the interpretation process: Does the instrument application process reinforce stigmas, does it have negative effects on the target populations? Do the evaluated characteristics reinforce problems or pathologies in these groups? Does the interpretation language present discriminatory biases? For psychology to contribute to knowledge construction in the 21 st century, it must be borne in mind that the investigation of its assumptions, regarding discriminatory biases, is an important step for psychological practice to serve everyone, regardless of their differences.

## Data Availability

No datasets were generated or analysed during the current study.

## Footnotes

1 It’s crucial to acknowledge findings advanced by researchers like Hollingworth and Woolley predominantly addressed the experiences of white, urban, upper-class women. Their analyses, while groundbreaking for their, did not adequately account for the distinct and often harsher realities faced by Black women and women from impoverished backgrounds (Hooks, 2000). The complex interplay of race, class, and gender — what we now understand as intersectionality. A more nuanced and inclusive understanding of women’s diverse conditions, recognizing the differential impact of intersecting oppressions, only began to gain significant theoretical and empirical traction in the 1960 s, marking a crucial shift in feminist scholarship and psychology.

2 Unfortunately, a recent and troubling trend has emerged within a fringe of the feminist movement, characterized by anti-trans sentiment that aligns with the moral agendas often seen on the far right (Tudisco, 2023). This development tragically diverges from the movement’s historical commitment to expanding liberation and challenging rigid gender norms for all.
